# Robust place recognition under illumination changes using pseudo-LiDAR from omnidirectional images

**DOI:** 10.1038/s41598-026-39848-y

**Published:** 2026-02-13

**Authors:** Juan José Cabrera, Marcos Alfaro, Arturo Gil, Oscar Reinoso, Luis Payá

**Affiliations:** 1 Institute for Engineering Research (I3E), Miguel Hernández University, Av. Universidad s/n, 03202 Elche, Comunidad Valenciana Spain; 2 Valencian Graduate School and Research Network for Artificial Intelligence (valgrAI), Valencia, Comunidad Valenciana Spain

**Keywords:** Place recognition, Point clouds, Data augmentation, Depth estimation, Omnidirectional vision, Engineering, Mathematics and computing, Optics and photonics

## Abstract

Visual Place Recognition (VPR) systems typically exhibit reduced robustness when subjected to changes in scene appearance produced by illumination dynamics or heterogeneity across different types of visual sensors. This paper proposes a novel framework that exploits depth estimation techniques to overcome these challenges. Our approach transforms omnidirectional images into depth maps using Distill Any Depth, a state-of-the-art depth estimator based on Depth Anything V2. These depth maps are then converted into pseudo-LiDAR point clouds, which serve as input to the MinkUNeXt architecture, which generates global-appearance descriptors. A key innovation lies in our novel data augmentation technique that exploits different distilled variants of depth estimation models to enhance robustness across varying conditions. Despite training with a limited set of images captured only under cloudy conditions, our system demonstrates robust performance when evaluated across diverse lighting scenarios, and further tests with different datasets and camera types confirm its generalization to geometrically dissimilar inputs. Extensive comparisons with state-of-the-art methods prove that our approach performs competitively across diverse lighting conditions, particularly excelling in scenarios with significant illumination changes. Furthermore, the generation of pseudo-LiDAR information from standard cameras provides a cost-effective alternative to 3D sensors. In summary, this work presents a fundamentally different approach to scene representation for VPR, with promising implications for robot localization in challenging environments. The implementation is publicly available at https://juanjo-cabrera.github.io/projects-pL-MinkUNeXt/.

## Introduction

Mobile robotics has seen significant advancements lately, driven by the need to develop autonomous systems capable of navigating and operating in varied and complex environments. The autonomous navigation of mobile robots implies the integration of sensors and algorithms to perceive, understand and interact with their surroundings. Advances in sensor technology have been crucial in the development of these systems, enabling greater precision and reliability.

One of the persistent challenges in mobile robotics is place recognition, which involves identifying specific areas in the environment for navigation and localization purposes. Current place recognition systems utilize a variety of sensors, such as LiDARs^1^ and cameras^2^, each with its own advantages and limitations. LiDAR systems are self-illuminated sensors, thus intrinsically invariant to light changes in the scene. LiDAR systems possess the ability to obtain a detailed and precise 3D point cloud of the scene at a high frequency. Compared to LiDAR systems, omnidirectional cameras are often more economical and, at the same time, provide great amount of data from the environment that include shape, texture and color. In addition, omnidirectional cameras are able to capture a full $360^{\circ }$ view of the environment surrounding the robot^3^. In contrast, the appearance of the image captured by a camera is altered significantly when confronted to changing natural light conditions, such as shadows, glares and other effects.

Given the advantages and drawbacks of the available sensors, researchers are exploring multi-sensor approaches, such as the combination of standard cameras with LiDAR^4^ or with infrared cameras^5^. Such approaches aim to combine the strengths of each technology, improving robustness under diverse illumination conditions and enhancing the overall effectiveness of autonomous systems in real-world applications. However, these solutions require sophisticated data fusion algorithms and increased computational resources to process and integrate information from multiple sensors. Additionally, combining various sensors can significantly raise the overall cost and complexity, posing challenges in terms of calibration, synchronization and real-time data processing.

In this context, the use of a single omnidirectional camera as the only source of information for place recognition is interesting: the sensory part of the robot is more economical and avoids using expensive LiDAR systems. In contrast, achieving an invariant description of the scene based uniquely on visual data is hard. To address this challenge, this paper proposes the use of pseudo-LiDAR point clouds^6^, which are generated from depth maps obtained through advanced depth estimation models. The proposed approach leverages the advantages of depth information to enhance robustness against changes in visual appearance, while maintaining a cost-effective and lightweight sensory setup.

In summary, this work bridges the gap between visual and structural place recognition by proposing a pipeline that transforms single panoramic images into rich pseudo-LiDAR representations using Distill Any Depth^7^. By embedding these point clouds with a sparse convolution network, the system effectively decouples the scene’s geometry from its visual appearance. The main contributions of this manuscript are:

- A robust pseudo-LiDAR VPR framework that effectively tackles Visual Place Recognition in challenging indoor scenarios by extending 2D panoramic views into 3D point clouds. This approach leverages structural consistency to overcome the limitations of purely visual methods in severe lighting changes (e.g., day-to-night shifts) and unseen environments.
- A set of augmentation techniques tailored to enhance the robustness of pseudo-LiDAR models. The core contribution, Distilled Depth Variations, simulates realistic depth estimation inconsistencies to enhance the place recognition performance against illumination changes.
- A solution to enhance sparse 3D point clouds with illumination-invariant cues, such as intensity gradients. This hybrid representation based on geometric data ensures the robustness of the method against lighting variations.
- A sensor-agnostic approach that enables successful generalization to different camera models (e.g., pinhole and panoramic), by simply replacing the inverse camera projection.

The rest of the manuscript is structured as follows. Section “Related works” reviews the state of the art in place recognition. In Section "Pseudo-LiDAR place recognition from omnidirectional views" the methodology employed in this paper is detailed. Section “Experiments” describes the experiments. Finally, Section “Conclusion” includes the conclusions and future work.

## Related works

This section reviews the state of the art of place recognition with deep learning techniques. In particular, several approaches that have employed deep neural networks with LiDAR sensors are analyzed, cameras or a combination of both. Besides, this section includes some recent models that estimate depth by relying solely on visual information.

### Place recognition

Place recognition has been a topic of research for several decades, but it was not until the last decade that some authors started to explore the use of deep learning techniques^8,9^. Within the image domain, many researchers started to fine-tune large and pretrained CNNs, including VGG16^10^ or ResNet^11^, for their downstream tasks. Nevertheless, NetVLAD^12^ was the first end-to-end method to train a CNN for visual place recognition (VPR). Subsequently, vision transformers revolutionized the way to extract features from images, giving place to ViT^13^, Swin-L^14,15^ or DINO^16,17^, among others. Nowadays, VPR approaches focus on designing efficient and scalable training techniques, e.g. CosPlace^18^, EigenPlaces^19^ or AnyLoc^20^, or developing feature aggregation methods to obtain robust descriptors, such as MixVPR^21^ or SALAD^22^. In addition, other papers have proposed solutions that exploit the properties of omnidirectional vision for VPR^23,24^. The results of the aforementioned methods are adversely affected when the scenes are captured with different illumination conditions or the images suffer from visual aliasing.

Besides, LiDAR sensors have become a popular choice to tackle place recognition^25^. In this scope, PointNetVLAD^26^ was the first neural network able obtain global descriptors from point clouds. From that moment on, other approaches developed more complex architectures, based on 3D convolutional networks, to address the same problem, such as MinkLoc3Dv2^27^ or MinkUNeXt^28^, or based on transformers^29,30^. Other approaches have explored the combination of visual and LiDAR data^31^. In particular, Zhao *et al.*^32^ and Zhou *et al.*^4^ make use of LiDAR sensors along with omnidirectional cameras. However, LiDAR are still expensive devices that could be avoided to reduce the total cost of the mobile platform.

### Depth estimation

Recent advancements in foundation models have enabled significant progress in various computer vision tasks. These large-scale models, trained on diverse and extensive datasets, have demonstrated remarkable abilities to transfer knowledge across multiple downstream tasks^33^, such as depth estimation, which consists in predicting the distance to the camera for every pixel of an image^34^. Depth estimation models can be classified into two groups: generative models^35,36^, which are able to model the details with a higher precision, and discriminative models^37,38^, which are more robust to changes in the scene. Depth Anything^39^ belongs to the second group and achieved the best results in the state of the art. Furthermore, the same authors have recently presented Depth Anything V2^40^, which has clearly outperformed their old version. Inspired on this paper, Hu *et al.*^41^ and subsequently Chen *et al.*^42^ have proposed video depth estimators, which ensure consistency in the depth prediction across consecutive frames. Also, Guo *et al.*^43^ have developed and trained a model with different kinds of images (standard, fisheye and equirectangular), with the aim of increasing the quality of depth maps regardless of the camera type.

Model distillation has contributed significantly to recent advances in depth estimation. This process consists in training smaller models by employing the largest model as the teacher, i.e., using the predictions of this model as labels to train the student models. In this sense, DAv2 presents a Giant model and three distilled versions (Base, Small, Large). Besides, He *et al.*^7^ have recently developed Distill Any Depth, which employs DAv2 as backbone and has been trained with multiple teachers, GenPercept^44^ and DAv2-Large, leading to a better performance than DAv2.

Due to the rise of depth estimation models in the recent years, other authors have sought to integrate predicted depth maps into their algorithms to address different downstream tasks, including object detection^45^, augmented reality^46^ or medical diagnosis^47^. Within place recognition, Hettiarachchi *et al.*^48^ have developed a two-branch architecture that processes RGB images and estimated depth maps, obtained with the ZoeDepth model^38^, in the 2D domain, and they evaluate their method with pinhole images, captured under daylight conditions. Therefore, the use of monocular omnidirectional depth estimation in order to obtain a pseudo-LiDAR representation to address VPR in the 3D domain and assess its impact on the illumination variance still remain unexplored.

Consequently, a novel place recognition method is presented in this paper, which aims to increase the robustness against lighting variations and other visual phenomena. The proposed solution employs panoramic images as the only source of information, from which depth maps are generated using Distill Any Depth^7^. Afterwards, these depth maps are transformed into pseudo-LiDAR 3D point clouds, which are processed with the MinkUNeXt architecture^28^ to obtain a global descriptor.

## Pseudo-LiDAR place recognition from omnidirectional views

**Fig. 1:**
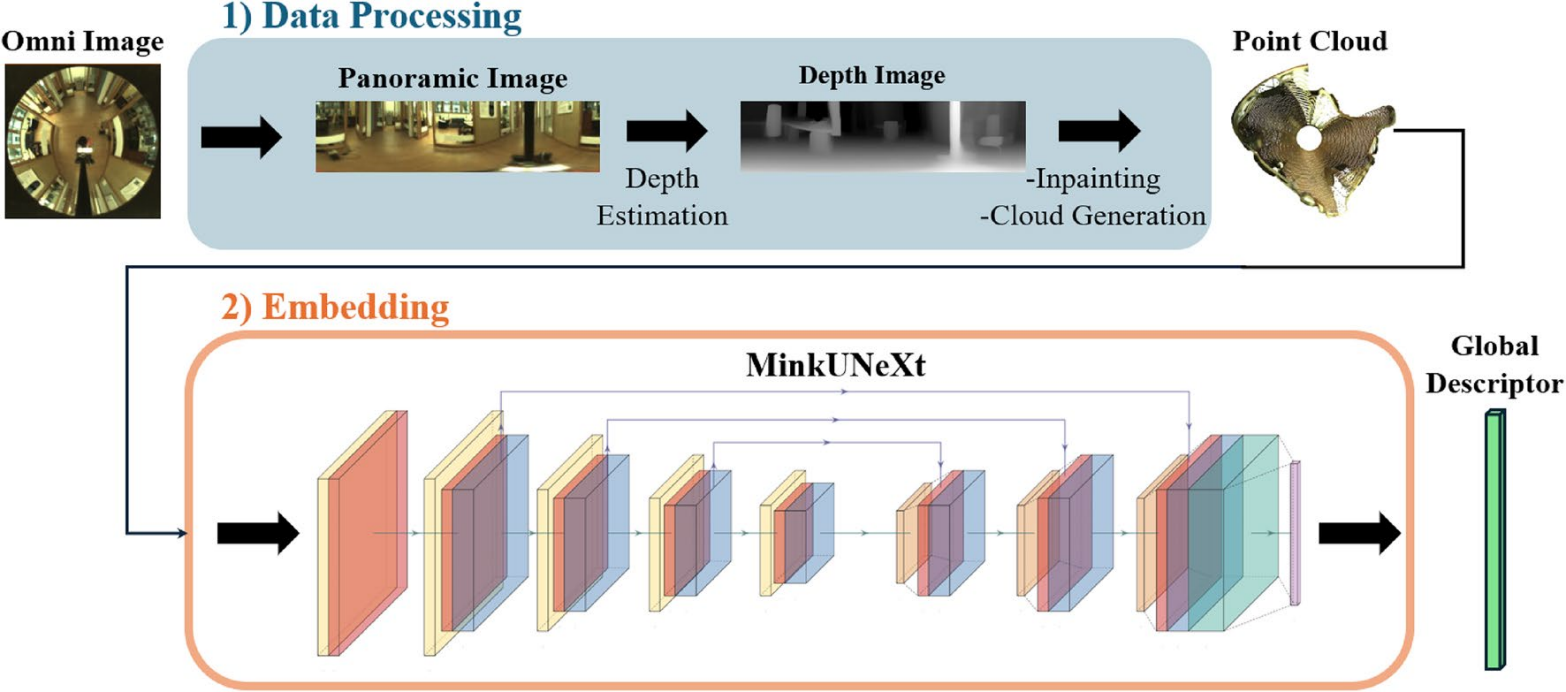
General outline of the method proposed in this paper, which consists of two steps: (1) The omnidirectional image is transformed into a 3D point cloud by means of a depth estimation map, obtained with Distill Any Depth^7^ and (2) The point cloud is embedded into a global descriptor with the MinkUNeXt architecture.

To address visual place recognition, this paper employs omnidirectional images captured in indoor environments using a catadioptric system mounted on a mobile robot. This system combines a hyperbolic mirror and a monocular camera to capture images with a field of view of $360^{\circ }$, enabling comprehensive visual data collection in a single frame. The images are converted afterwards into a panoramic format with a resolution of 128x512x3 pixels (RGB), providing a wide horizontal field of view suitable for indoor navigation tasks. These panoramic images are then transformed into depth images using the Distill Any Depth^7^. The complete method is detailed in Fig. 1. The rest of the proposal is summarized in the following subsections.

### Depth estimation

Distill Any Depth^7^ is based on the Depth Anything V2 model^40^, a state-of-the-art depth estimation architecture designed to generate precise depth maps from monocular images. This model is built upon the DINOv2^17^ backbone, a vision transformer architecture renowned for its ability to capture relationships between distant elements in an image and contextual information. These features enable highly detailed and accurate depth estimations, even in challenging indoor environments. The Distill Any Depth model is trained using a multi-teacher distillation framework, which integrates the strengths of multiple teachers, that is GenPercept^44^ and DAv2^40^, while combining local and global depth features to enhance the student model’s predictions.

**Fig. 2:**
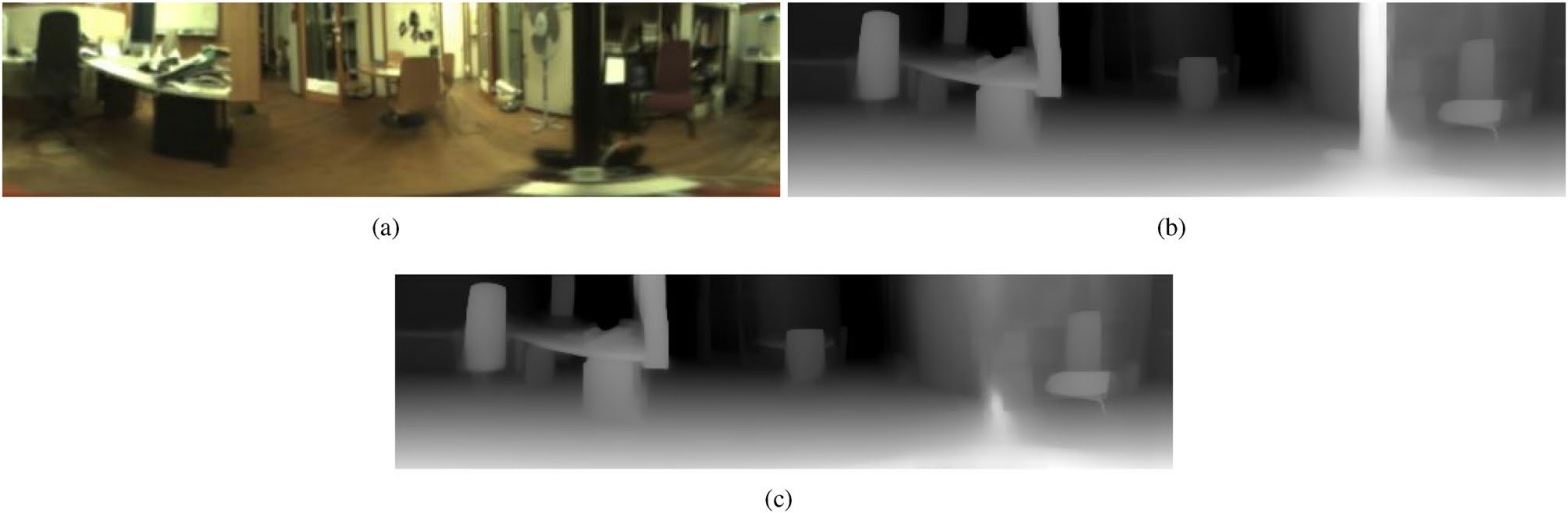
Examples of (**a**) an omnidirectional image from the COLD database converted into a panoramic format, (**b**) a depth map obtained with Distill Any Depth from the panoramic image and (**c**) a depth map after inpainting with LaMa.

The depth estimation model processes the panoramic images to infer a pixel-wise depth map, effectively converting the 2D panoramic image into a corresponding depth representation. Each pixel in the depth map encodes the relative distance from the camera to the observed scene, providing a detailed spatial understanding of the environment. These depth maps serve as the foundation for generating three-dimensional point clouds, which are later utilized for robust place recognition. Figure 2 illustrates an example of a depth map generated using Distill Any Depth from a panoramic image.

### Depth post-processing

Since the omnidirectional catadioptric system consists of a monocular camera paired with a hyperbolic mirror, the structure that supports the mirror consistently appears as an artifact in the depth images, producing occlusions. To increase the robustness of the depth images to rotations and occlusions, inpainting techniques such as LaMa^49^ are employed.

LaMa is a state-of-the-art inpainting model that uses Fourier convolutions to reconstruct missing or occluded areas in images, effectively filling gaps with realistic and contextually appropriate data. In the current approach, LaMa is used to reconstruct the depth values in areas occluded by the structure that supports the hyperbolic mirror, thereby enhancing the overall completeness and accuracy of depth data. The application of LaMa to the depth map minimizes the impact of visual artifacts and ensures that the point clouds generated from the images are visually coherent and robust.

This post-processing step is critical for maintaining the reliability of the place recognition system, especially in dynamic indoor environments where lighting and objects positions can frequently change. The inpainted depth maps provide a more accurate basis for generating 3D point clouds, which will be subsequently used for the place recognition task. Figure 2 (b) and (c) show an example of a depth map before and after performing the inpainting operation with LaMa, respectively.

### Point cloud estimation

The depth information $d$ obtained from the Distill Any Depth model is initially represented as a dimensionless quantity in the range of 0 to 255. As a result, each pixel is associated to an estimated depth distance. These values are then transformed into depth measurements $d_{m}$ in meters using the following equation:

1 $$\begin{aligned} d_{m} = d_{min} + d\cdot d_{s} \end{aligned}$$

where $d_{min}$ and $d_{s}$ are the assumed minimum distance and depth factor scale, respectively. To obtain the point cloud, first, each pixel in the panoramic image is mapped to its cylindrical coordinates ($d_m$, $\theta$, *z*), where the radial distance for each pixel is $d_m$ and the azimuth angle $\theta$ and height *z* are calculated with eq. 2:

2 $$\begin{aligned} \theta = \frac{u}{w} \cdot 2\pi , \ \ \ z = \left( v - \frac{h}{2}\right) \cdot h_{s}, \end{aligned}$$

where $u$ is the horizontal pixel position and $w$ is the image width, $v$ is the vertical pixel position, $h$ is the image height and $h_{s}$ is the vertical scale factor. Next, the cylindrical coordinates are converted to Cartesian coordinates $(x, y, z)$ using the following equation:

3 $$\begin{aligned} y = d_{m} \cdot \sin (\theta ), \quad x = d_{m} \cdot \cos (\theta ), \quad z=z \end{aligned}$$

**Fig. 3:**
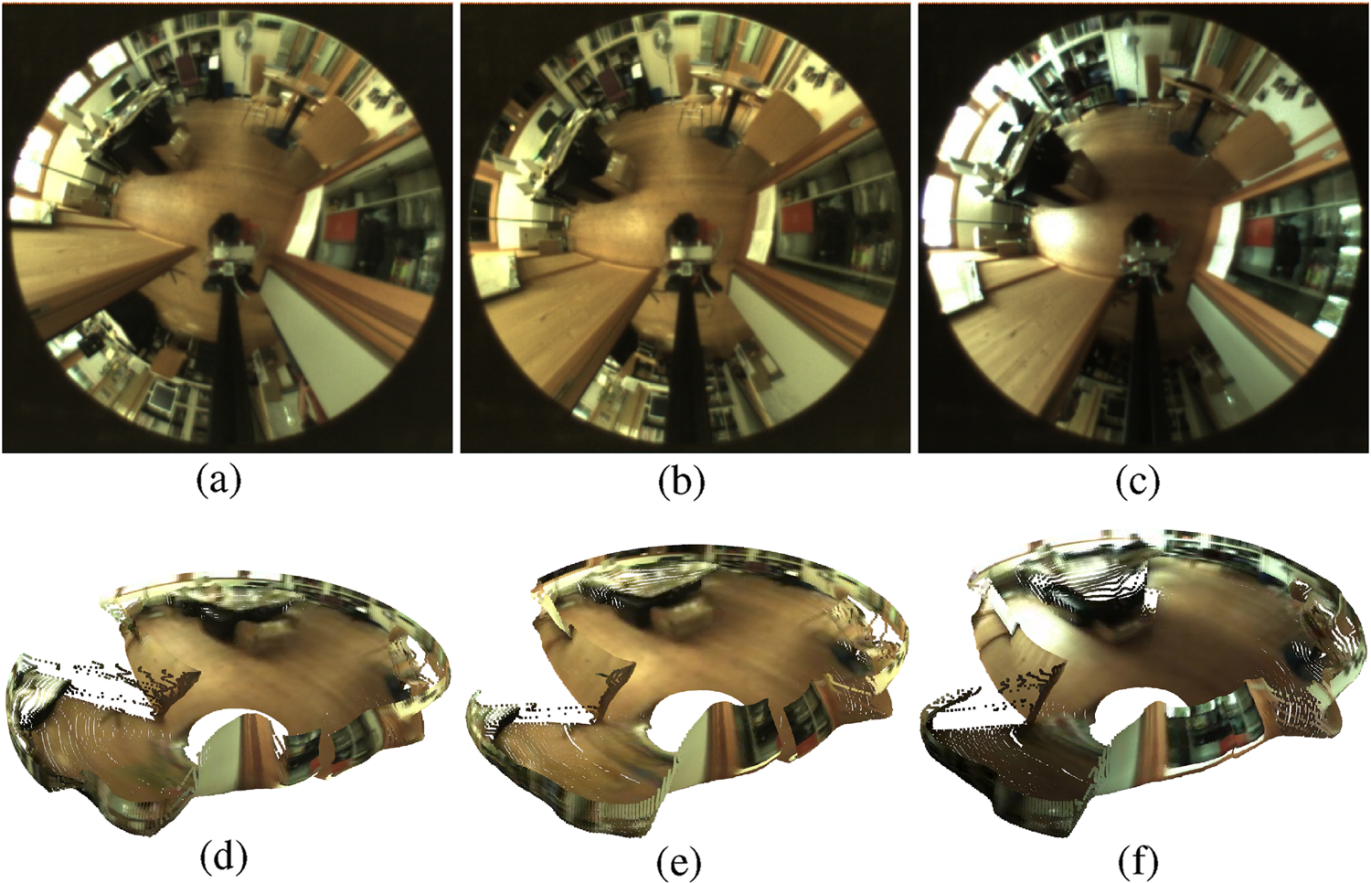
Omnidirectional images (**a**, **b**, **c**) captured under cloudy, night and sunny conditions, respectively and their estimated point clouds (**d**, **e**, **f**), obtained from the depth images.

The point cloud obtained from every omnidirectional image represents a three-dimensional spatial structure of the environment, providing critical distance information that enhances the robot’s ability to recognize places accurately. Table 2 contains the parameters values used to generate the point clouds. Besides, Fig. 3 includes three omnidirectional images captured under different lighting scenarios and the point cloud estimated for each of them. The three images are captured from, approximately, the same position and orientation, and, as a result, the estimated point clouds should be equal. However, the different illumination conditions alter the depth estimation. In the current approach, we aim to improve the resilience of the place recognition model against these effects by training it with a specifically designed data augmentation technique, which is detailed in the following subsections.

### Point cloud feature extractor and embedding

Place recognition from point clouds can be approached as an embedding task, where the goal is to extract the most descriptive features of a scene and aggregate them into a single vector descriptor that best represents the scene’s information.

For this purpose, MinkUNeXt^28^ is trained for both feature extraction and fusion. The extraction of features is conducted by a u-shaped encoder-decoder architecture, while the aggregation of these features into a single descriptor is handled by a Generalized Mean Pooling (GeM) layer^50^. MinkUNeXt, specifically designed for place recognition in outdoor environments, employs 3D Sparse Convolutions^51^ which makes it particularly well-suited for processing point clouds with sparse input information.

The input to the MinkUNeXt model is a point cloud represented as an unordered set of 3D coordinates $P = \{(x_i, y_i, z_i)\}$. This point cloud is quantized into a sparse tensor, which extends the concept of a sparse matrix to higher dimensions, with non-zero elements represented by a set of coordinates *C* and their associated features *F*. These features can be derived from the input image (e.g., RGB, grayscale, hue, etc.), from the point cloud coordinates (e.g., xyz coordinates, normals, etc.), or initialized to ‘ones’ to allow the model to learn the most suitable features given the input point cloud.

The proposed approach consists in feeding the MinkUNeXt model with the gradients of the image as features. The gradient is computed using a Sobel operator applied on the intensity image with a 3x3 kernel. The resulting gradient image highlights areas of high spatial frequency, often associated with edges and textures in the image.

The gradient is then decomposed into magnitude and direction, where the magnitude represents the strength of the gradient at each pixel, and the direction indicates the orientation of the gradient. The direction is projected onto the unit circle using sine and cosine functions, enabling a compact and differentiable representation of the gradient’s orientation.

The magnitude and direction of the gradient are concatenated afterwards to form a feature vector for each point in the point cloud. This feature vector captures both the strength and orientation of the gradient at each pixel, providing valuable data about the local structure and texture of the image. By incorporating this information into the point cloud features, the model’s ability to recognize places based on both geometric and visual characteristics is enhanced. Other visual features can also be associated to each point in the point cloud, such as, hue, saturation, intensity, etc. A study has been conducted and the results are detailed in the experimental section.

**Fig. 4:**
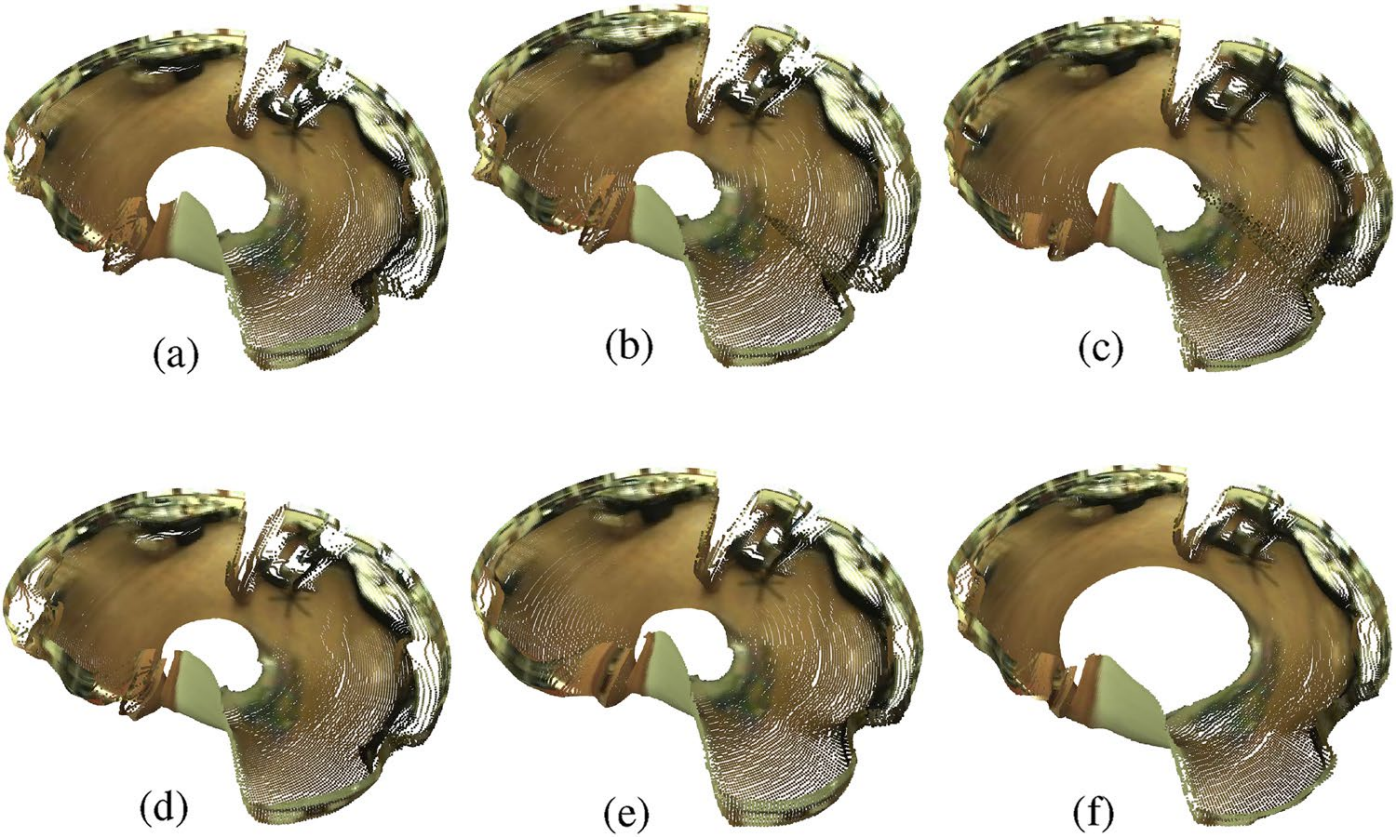
Example of Distilled Depth Variations effect applied to a cloudy image. (**a**) Distill Any Depth Large, (**b**) Distill Any Depth Base, (**c**) Distill Any Depth Small (**d**) Depth Anything Large, (**e**) Depth Anything Base and (**f**) Depth Anything Small.

### Data augmentation

In this research work, a novel data augmentation technique called Distilled Depth Variations is presented, which is specifically designed to enhance the robustness of sparse 3D models when processing pseudo-LiDAR point clouds. This technique is complemented by established point cloud augmentation methods adapted for our pseudo-LiDAR approach. Experimental evidence shows that depth estimators are sensitive to environmental factors such as lighting conditions, leading to inconsistent predictions. To mitigate this effect, our primary augmentation strategy simulates realistic depth estimation variations, thereby improving model generalizability across different lighting conditions. Additionally, we introduce two geometric augmentation techniques: Remove Block and Radial Block Translation, specifically tailored to handle occlusions and depth artifacts. For a comprehensive evaluation, this data augmentation approach is compared with conventional techniques commonly used in point cloud processing, which include:

- **Point Dropout**: This technique randomly eliminates a subset of points (e.g., eliminating a 20% of the points of the cloud) to simulate partial occlusions or sensor dropouts. It encourages the model to rely on contextual and structural features rather than overfitting to specific point distributions.
- **Rotation**: The cloud is rotated around the vertical axis (yaw) by a random angle (e.g., in the range $[-180, +180] ^{\circ }$), simulating viewpoint changes that are common in autonomous navigation as the robot moves through the environment and changes its orientation. This enhances orientation invariance while preserving elevation angles critical for LiDAR-based perception.
- **Remove Block**: Introduced in this research, this technique removes a cuboid region of variable size (e.g., 2–50% of the cloud’s volume) from the point cloud. Unlike point dropout, this technique simulates large occlusions, training the model to handle significant missing data regions.
- **Radial Block Translation**: Also proposed in this study, this method translates a cuboid region of variable size (e.g., 2–30% of the cloud’s volume) along the radial axis relative to the center of the point cloud. This technique is specifically designed to address depth prediction variations caused by differing illumination conditions.
- **Planar Scaling**: The point cloud is uniformly scaled by a factor (e.g., scaling by a factor varying from 0.8 to 1.2) along the *x* and *y* axes to emulate depth estimation changes or objects size variations. This technique also addresses depth prediction variations under varying illumination conditions.
- **Elastic Distortions**: This augmentation technique applies random elastic deformations to the point cloud, simulating non-rigid transformations that may occur in real-world scenarios. The method generates a smoothed Gaussian noise grid, which is interpolated and applied to the spatial coordinates of the point cloud.
- **Distilled Depth Variations**: This is the core novel technique proposed in this paper, which selectively estimates depth using different distilled versions of DAv2 (small, base, large) and Distill Any Depth (small, base, large) - see Fig. 4. Unlike techniques such as Radial Block Translation or Planar Scaling, this method introduces depth distortions based on the predictions of less robust models (e.g., the small and base variants). By simulating the inaccuracies of weaker depth estimators, this approach enhances the model’s resilience to depth estimation errors inherent in pseudo-LiDAR generation pipelines.

## Experiments

### Datasets

The images used in this paper belong to the COLD dataset^52^, which can be downloaded from (https://www.cas.kth.se/COLD/). This dataset is composed of several indoor environments: Freiburg A & B (FR-A, FR-B) and Saarbrücken A & B (SA-A, SA-B). In each environment, a mobile robot follows different paths and captures omnidirectional images with a catadioptric system. These images have been captured under three different lighting conditions: cloudy, night and sunny. Besides, the images contain people moving and changes in the position of the objects from the scene. Overall, this dataset poses a variety of challenging conditions that effectively validate our method.

Table 1 shows the number of images that compose the training, database and evaluation sets. The MinkUNeXt model has been trained only with 4338 images captured under cloudy scenarios from the Freiburg A set (FR-A * seq2_cloudy1* and *seq2_cloudy3*). The other scenarios and lighting conditions are used to evaluate the robustness and the performance of our pipeline: FR-A (*seq2_cloudy2*, *seq2_night2*, *seq2_sunny2*), FR-B (*seq3_cloudy2*, *seq3_sunny2*), SA-A (*seq2_cloudy2*, *seq2_night1*) and SA-B (*seq4_cloudy2*, *seq4_night2*, *seq4_sunny1*). The database consist of cloudy images of each environment and it is obtained by downsampling consecutive frames to ensure an average distance of 20 cm between capture points. The specific sequences chosen for the databases are FR-A *seq2_cloudy3*, FR-B *seq3_cloudy1*, SA-A *seq2_cloudy3* and SA-B *seq4_cloudy1*.

**Table 1:** Number of training and evaluation queries of the different scenarios for the three lighting conditions.

	Train	Database	Evaluation		
	Cloudy	Cloudy	Cloudy	Night	Sunny
FR-A	4338	556	2595	2707	2114
FR-B	-	560	2008	-	1797
SA-A	-	586	2774	2267	-
SA-B	-	321	836	870	872

### Labeling and similarity

Each image in the dataset is annotated with its pose data, which serves as ground truth for the robot’s path. The pose acts as a unique identifier for each image and is used as labels in the training and as ground truth to evaluate the performance of the place recognition method.

Next, the concept of similarity between pseudo point clouds obtained from images is defined. This concept is crucial for place recognition since the model needs to be trained with structurally similar point clouds captured from the same location, as well as structurally dissimilar point clouds captured from different locations. Most similarity protocols are based on the Euclidean distance between the coordinates from which the point clouds were captured. Two point clouds are considered similar if captured within a distance *p* and dissimilar if captured from a distance greater than *n* (where $p \le n$). This approach assumes that samples captured from the same vicinity will share a similar appearance, making it a straightforward but effective method for training.

### Implementation details

In this research, the MinkUNeXt model is trained according to the procedure established in^28^. This process utilizes the Truncated Smooth-AP loss function $\mathscr {L}_{TSAP}$, which aims to maximize the ranking of the top-k positive candidates:

4 $$\begin{aligned} \mathscr {L}_{TSAP} = \frac{1}{b} \sum _{q=1}^{b}(1 - AP_q) \end{aligned}$$

where *b* is the batch size and $AP_{q}$ represents the smooth average precision:

5 $$\begin{aligned} AP_q = \frac{1}{|P |}\sum _{i \in P} \frac{1 + \sum _{j \in P, j \ne i} G(d(q,i) - d(q,j); \tau )}{1 + \sum _{j \in \Omega , j \ne i} G(d(q,i) - d(q,j); \tau )} \end{aligned}$$

The computation of the average precision $AP_q$ for a query point cloud *q* involves the soft ranking of the *k* most similar positive examples *P* and all the available positive and negative instances $\Omega$. This calculation employs a sigmoid function $G(x;\tau ) = \left( 1 + \exp \left( -\frac{x}{\tau }\right) \right) ^{-1}$ to create a differentiable approximation of traditional ranking, where the sigmoid temperature $\tau$ controls the steepness of the ranking transition. Moreover, the Euclidean distance *d*(*q*, *i*) quantifies the dissimilarity between *q* and any point cloud *i* in the dataset.

To perform effectively, this loss function needs a large batch size. In the experiments, a batch size of 512 was used, and the AdamW optimizer was employed to minimize the loss. Furthermore, the ablation study is conducted by training the MinkUNeXt model during 50 epochs with scheduler steps in epochs 20 and 30. In contrast, for the comparison with the state-of-the-art, the model is trained for 200 epochs with scheduler steps in epochs 150 and 180, to ensure optimal performance. The experiments are conducted using an NVIDIA GeForce RTX 3090 GPU equipped with 24 GB of memory. The parameters and values used for generating the point clouds and training the model are summarized in Table 2.

**Table 2:** Point cloud generation and training parameters.

Parameter	Value
Depth min. distance ($d_{min}$)	1.0 m
Depth scale factor ($d_{s}$)	0.002 m/pixel
Vertical scale factor ($h_{s}$)	0.015 m/pixel
Positive distance (*p*)	0.4 m
Negative distance (*n*)	0.4 m
Batch Size (*b*)	512
Number of Epochs	200 (50)
LR Scheduler Steps	150, 180 (20, 30)
Initial Learning Rate	$1 \times 10^{-3}$
L2 Weight Decay	$1 \times 10^{-4}$
Sigmoid Temperature ($\tau$)	0.01
Positives per Query (*k*)	16
Quantization Scale (*qs*)	0.01
Distance threshold (*d*)	0.5

The performance of place recognition is evaluated using the Recall at *N* (R@N) metric with a distance threshold of *d* meters. This metric is defined as the percentage of queries for which at least one of the top *N* predictions is within *d* meters of the query location. Formally, it is expressed as:

6 $$\begin{aligned} \text {R@}N = \frac{|\{q \in Q \mid \exists p \in P_q^N \text { such that } d(p, q) \le d\}|}{|Q|} \end{aligned}$$

where *Q* is the set of all query images, $P_q^N$ represents the top *N* predicted locations for query *q*, and *d*(*p*, *q*) denotes the Euclidean distance between the predicted location *p* and the ground truth location of query *q*.

In this paper, two variants of this metric are assessed: R@1 and R@1%. For R@1, we set $N = 1$, meaning that a query is considered correctly recognized if its top-ranked prediction is within the *d*-meter threshold. For R@1%, we define *N* as the number of point clouds corresponding to the top 1% of the total reference database, allowing evaluation over a broader set of predictions.

### Ablation study

This section studies different state-of-the-art depth estimators, the effect of the proposed data augmentation technique for pseudo-LiDAR and the selection of the input features to feed the MinkUNeXt model.

#### Depth estimators

In this experiment, state-of-the-art depth estimators and their distilled models are employed to generate depth maps from panoramic images. These depth maps are subsequently converted to pseudo-LiDAR point clouds, which are used to train the MinkUNeXt model for the VPR task. The training set is composed of 4338 point clouds generated from cloudy images that were captured in Freiburg Part A environment (FR-A). Table 3 contains the evaluation of the place recognition task in terms of R@1 with each depth estimator under different environments (FR-A, FR-B, SA-A and SA-B) and lighting conditions (cloudy, night and sunny).

**Table 3:** Ablation study of different depth estimation models in all the environments in terms of R@1.

Depth Estimator (R@1)	Freiburg-A			Freiburg-B		Saarbrücken-A		Saarbrücken-B			
	Cloudy	Night	Sunny	Cloudy	Sunny	Cloudy	Night	Cloudy	Night	Sunny	Global
DepthPro^53^	80.25	77.70	60.22	68.13	68.73	60.05	50.89	69.14	60.34	62.84	65.83
DAv2 (Small)^40^	89.64	92.21	79.94	82.52	89.32	71.77	64.87	84.33	74.71	81.65	81.10
DAv2 (Base)^40^	89.52	89.61	78.95	81.82	**91.04**	70.94	68.95	83.61	78.05	79.70	81.22
DAv2 (Large)^40^	**91.42**	94.32	86.28	**83.96**	87.37	75.82	71.62	86.84	78.62	**81.65**	83.79
Distill Any Depth (Small)^7^	90.26	90.46	76.92	81.82	86.48	71.59	63.79	83.13	70.34	77.29	79.21
Distill Any Depth (Base)^7^	91.19	93.32	84.34	83.72	90.26	**75.91**	**73.59**	83.13	75.17	80.73	83.14
Distill Any Depth (Large)^7^	91.00	**94.92**	**88.41**	**83.96**	86.64	75.63	70.59	**89.23**	76.67	81.31	**83.84**

Regarding the comparison of the state-of-the-art depth estimators (Table 3), a clear distinction in performance is observed based on model scale and architecture. While DAv2-L^40^ outperformed other models under the cloudy conditions of FR-A, which is the lighting condition employed both as training and database information, the Distilled Any Depth^7^ family demonstrated superior versatility. When considering the global performance across all datasets and weather conditions, Distill Any Depth (Large) emerges as the most robust estimator with a global R@1 of 83.84%, marginally surpassing DAv2 (Large) at 83.79%. In comparison, other models such as DepthPro^53^ or the smaller variants of DAv2 (Small) and Distilled Any Depth (MT-Small) showed competitive but lower recall, particularly in challenging lighting conditions.

Based on these results, Distilled Any Depth MT-Large was selected as the model for estimating pseudo-LiDAR data, owing to its superior accuracy and robustness across diverse environments and lighting conditions. This choice ensures reliable depth estimation, which is critical for the success of the downstream task of place recognition.

#### Data augmentation

Regarding data augmentation, we evaluate three proposed strategies: two geometric effects (*Remove Block* and *Radial Block Translation*) and one depth-driven approach (*Distilled Depth Variations*). These are compared against the baseline and standard techniques like *Rotation* and *Point Dropout*. Table 4 details the R@1 metric across diverse environments.

First, the baseline model (without data augmentation) exhibited considerably high R@1 value across all conditions, with a slight degradation in accuracy under SA-A night condition. This highlights the lack of robustness of depth estimator models when varying lighting conditions, particularly when the database and query images are subject to significant illumination shifts.

Among the proposed geometric strategies, the Radial Block Translation proved to be the most effective structural augmentation, achieving a Global R@1 of 84.11%. Additionally, the Remove Block effect offered slight global improvements over the baseline, outperforming classic methods like Rotation, which produced a better performance of MinkUNeXt under cloudy conditions of FR-A and FR-B, but degraded the overall performance to 82.04%. In contrast, the proposed Distilled Depth Variations (the core augmentation effect proposed in this study) achieved the best robustness with a Global R@1 of 85.34% (+ 1.5%). Unlike the geometric proposals, this method leverages depth inconsistencies, endowing the MinkUNeXt model with a better generalization. Crucially, it improved the recall in Saarbrücken-A Night by +3.38% and Saarbrücken-B Night by +5.51% compared to the baseline. This confirms that while geometric augmentations (Radial/Remove Block) are useful, distilling depth variations provides the necessary adaptability for unconstrained lighting conditions.

**Table 4:** Evaluation of the Data Augmentation effects in all the environments in terms of R@1.

Augmentation Effect (R@1)	Freiburg-A			Freiburg-B		Saarbrücken-A		Saarbrücken-B			
	Cloudy	Night	Sunny	Cloudy	Sunny	Cloudy	Night	Cloudy	Night	Sunny	Global
Baseline	91.00	94.92	88.41	83.96	86.64	75.63	70.59	89.23	76.67	81.31	83.84
Point Dropout	91.31	94.92	87.37	82.82	84.53	74.99	72.33	87.44	76.90	80.62	83.32
Rotation	**91.89**	94.84	86.28	**85.86**	85.64	75.17	68.67	83.85	72.53	75.69	82.04
Remove Block	90.45	94.55	86.71	84.66	89.26	75.86	72.09	87.56	75.75	**82.80**	83.97
Radial Block Translation	91.00	**95.44**	87.94	83.27	**89.71**	**76.14**	69.61	88.28	77.47	82.22	84.11
Planar Scaling	91.23	95.18	87.56	83.32	86.81	75.08	69.70	89.00	78.62	80.50	83.70
Elastic Distortions	90.96	95.32	86.00	85.11	88.20	76.00	68.34	83.49	75.17	80.28	82.89
**Distilled Depth Variations (ours)**	91.62	94.84	**89.45**	85.36	87.15	75.49	**73.97**	**90.79**	**82.18**	82.57	**85.34**

#### Input features

This section analyzes the input features associated to the 3D coordinates of each point, Table 5 presents the evaluation of different point cloud features across various lighting conditions and environments. The features employed in this experiment are related to color information (RGB, gray scale and hue) or intensity data (gradient).

The results demonstrate the impact of feature selection on the robustness and accuracy of the place recognition model, particularly under challenging lighting scenarios. In general terms, when visual features (RGB and gray scale) were added to the point cloud, the model specially achieved strong performance when tested under the same lighting conditions that conformed the map (database). However, the performance often degraded under night conditions, especially for RGB, highlighting the limitations of color features in handling bright and overexposed images. In contrast, the use of some particular features, such as hue or gradient, led to more consistent results, especially under night scenarios.

As a general rule, using the gradient features (both magnitude and argument) led to the best balance across the three different illuminations. Consequently, this experiment was extended to 200 epochs, whereas the rest of the experiments consisted of 50 epochs. In this case, a significant improvement was observed in global terms (+1.97%) and in particular, under FR-B sunny (+4.17%) and SA-B sunny (+5.73%), with respect to the model trained without visual features.

**Table 5:** Evaluation of different input features in all the environments in terms of R@1.

Point cloud features (R@1)	Freiburg-A			Freiburg-B		Saarbrücken-A		Saarbrücken-B			
	Cloudy	Night	Sunny	Cloudy	Sunny	Cloudy	Night	Cloudy	Night	Sunny	Global
Non-visual info.	91.62	94.84	89.45	85.36	87.15	75.49	73.97	90.79	82.18	82.57	85.34
RGB	92.20	94.29	86.14	**85.91**	90.65	76.51	57.65	83.25	71.15	81.88	81.96
Gray Scale	92.05	95.84	89.74	85.41	89.04	75.22	61.35	84.81	79.54	82.11	83.51
Hue	91.93	94.88	88.69	84.91	87.65	76.18	75.19	91.03	81.49	83.14	85.51
Gradient (Mag.)	91.73	95.18	89.12	85.01	87.53	75.59	**76.22**	90.43	80.57	83.72	85.51
Gradient (Arg.)	93.13	95.36	88.46	83.86	89.04	77.89	72.94	88.88	74.71	85.32	84.96
Gradient (Mag., Arg.)	92.82	**95.99**	89.36	85.56	87.26	78.71	74.77	90.55	80.00	87.73	86.27
^+^Gradient (Mag., Arg.)	**94.49**	95.92	**90.73**	85.71	**91.32**	**79.13**	71.25	**93.18**	**83.10**	**88.30**	**87.31**

^+^ Results obtained for 200 epochs.

### Evaluation with pinhole images

**Fig. 5:**
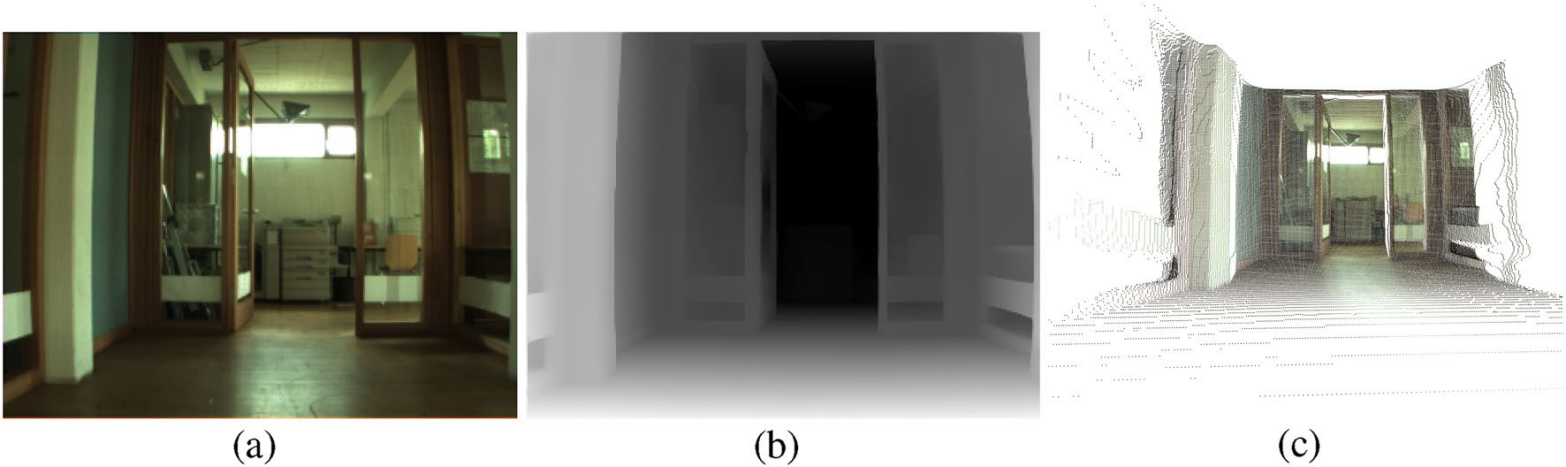
Examples of (**a**) a pinhole image from the COLD dataset, (**b**) a depth map obtained with Distill Any Depth from the standard image and (**c**) point cloud obtained with the inverse pinhole projection.

**Fig. 6:**
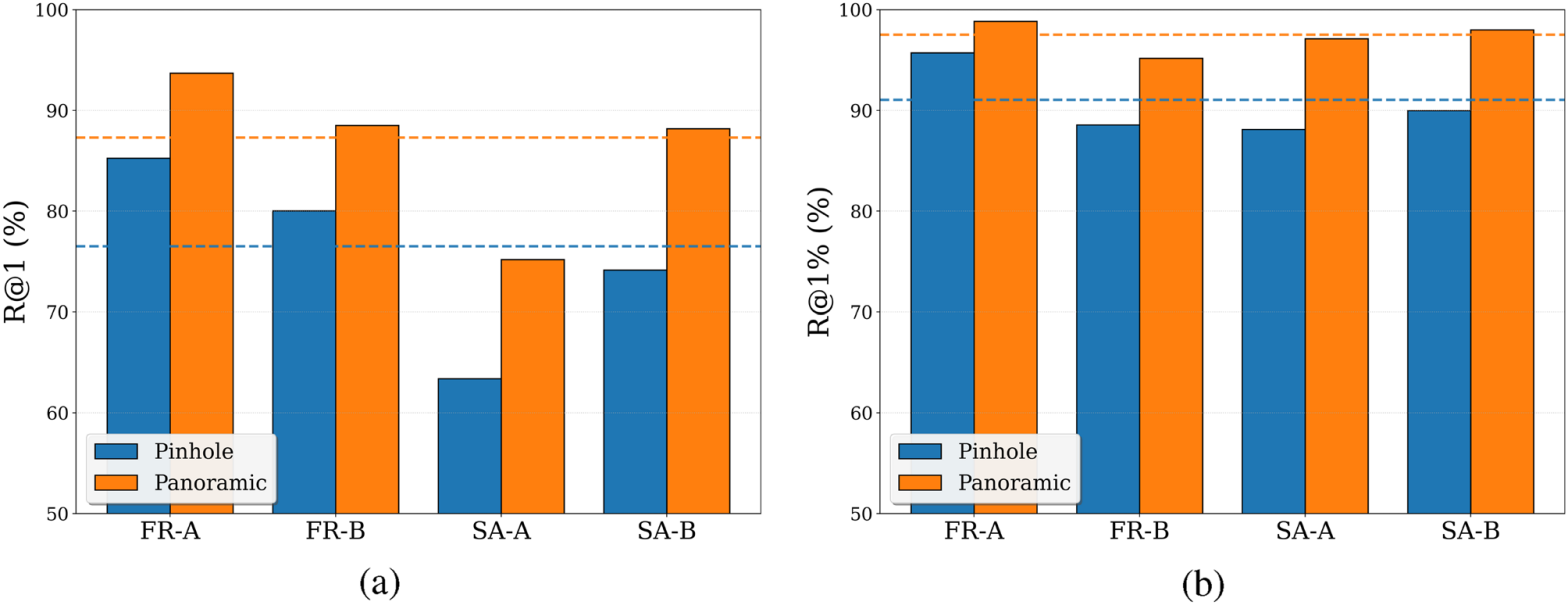
Performance of pL-MinkUNeXt with pinhole and panoramic images, in terms of (**a**) R@1 (**b**) R@1%.

Although pL-MinkUNeXt has been designed and exhaustively tested with omnidirectional images, it is not limited to this kind of optical system. To demonstrate this fact, the proposed method is evaluated with pinhole images from the COLD dataset. The adaptation is straightforward, as it is only necessary to replace the inverse cylindrical projection with the inverse pinhole projection. The optimal configuration of our method (obtained in the previous ablation study) is employed to fine-tune the MinkUNeXt model by using the point clouds obtained from the pinhole images. Figure 5 contains a sample pinhole image from the COLD dataset and its corresponding depth map and point cloud. Moreover, in Fig. 6, the performance of pL-MinkUNeXt with pinhole and panoramic images is compared.

The results presented in Fig. 6 confirm a fairly competitive performance of pL-MinkUNeXt with point clouds generated from pinhole images, achieving a 76.50% R@1 and a 91.05% R@1%. As expected, these values are lower than the ones obtained with panoramic images, since the overlapping field of view between the query and database pinhole images depends on the robot’s yaw angle, thus may be low in some cases, compared to the omnidirectional setup. While panoramic sensors capture a $360^{\circ }$ view of the environment, inherently preserving scene overlap regardless of the robot’s orientation (yaw), pinhole cameras are restricted to a narrow sector. Consequently, even moderate rotations or lateral translations can cause distinctive geometric features to disappear from the frame, drastically reducing the structural correspondence between the generated pseudo-LiDAR point cloud and the map. Despite these physical limitations, the method demonstrates remarkable generalization capability, proving that the proposed pseudo-LiDAR pipeline is sensor-agnostic and remains effective even with limited spatial context.

### Comparison with the state-of-the-art

To evaluate the quality of the method proposed in this paper, it is compared with both previous approaches that have used the COLD database and current state-of-the-art VPR methods. Tables 6 and 7 present the R@1 and R@1% metrics, respectively, for each technique across all environments and illumination conditions. The best result in each column is highlighted in bold, while the second-best result is underlined.

As evidenced in Table 6, pL-MinkUNeXt achieves the highest overall performance, reaching a global R@1 of 87.31%. This represents a substantial improvement over the best-performing 2D baseline (MixVPR^21^, 85.16%) and significantly outperforms other 3D-based approaches like MinkLoc3Dv2^27^ (84.07%) and CASSPR (82.70%)^54^.

Crucially, the benefits of the proposed pseudo-LiDAR approach are most prominent in challenging lighting conditions. While standard 2D methods such as CosPlace^18^ or EigenPlaces^19^ experience severe degradation in the Saarbrücken-A Night scenario (dropping to $\approx$62–65%), our method maintains a robust accuracy of 71.25%. This validates the hypothesis that structural depth features provide superior invariance to illumination changes compared to purely visual descriptors, which are prone to vanish in low-light environments. Furthermore, in the unseen Saarbrücken-B dataset, our method demonstrates remarkable generalization capabilities, leading the night results with 83.10% compared to the 73.68% of CASSPR^54^.

Regarding the R@1% metric (Table 7), pL-MinkUNeXt consistently achieves the top results, with a Global score of 97.52%. It is worth noting that in specific scenarios (e.g., Freiburg-B Sunny), some 2D methods like AnyLoc^20^ achieved slightly higher recall (98.05% vs. 97.89%). However, the proposed method based on pseudo-LiDAR offers the most balanced and robust solution for long-term operation across varying domains.

**Table 6:** Comparison with other VPR methods at different environments in terms of R@1.

Method (R@1)	Type	Freiburg-A			Freiburg-B		Saarbrücken-A		Saarbrücken-B			
		Cloudy	Night	Sunny	Cloudy	Sunny	Cloudy	Night	Cloudy	Night	Sunny	Global
Siamese VGG16^55^	2D	53.80	57.30	36.90	51.29	41.90	32.10	16.90	50.60	42.30	47.80	43.09
Triplet VGG16^24^	2D	83.70	83.20	61.00	55.60	48.20	33.70	17.60	38.50	32.60	51.40	50.55
AnyLoc^20^	2D	89.40	92.46	78.67	84.16	90.93	73.79	66.03	88.28	78.97	83.94	82.66
SALAD^22^	2D	90.64	90.80	80.13	84.46	90.71	75.59	66.83	85.65	82.64	84.17	83.16
CosPlace^18^	2D	91.64	92.21	79.99	**85.76**	92.60	76.56	62.68	91.39	80.57	86.58	84.00
Eigenplaces^19^	2D	92.22	93.02	81.41	85.31	**93.16**	77.92	64.31	91.75	80.23	86.12	84.55
CricaVPR^56^	2D	90.29	93.35	82.78	84.46	**93.16**	75.77	71.02	91.87	81.26	84.75	84.87
MixVPR^21^	2D	90.98	93.50	83.30	84.01	92.82	76.56	70.53	91.15	81.61	87.16	85.16
MinkLoc3Dv2^27^	3D	90.73	95.36	85.15	84.36	89.98	76.92	70.12	87.56	78.05	82.45	84.07
CASSPR^54^	3D	91.42	94.62	84.44	85.20	86.25	77.29	68.67	85.41	73.68	80.05	82.70
**pL-MinkUNeXt (ours)**	3D	**94.49**	**95.92**	**90.73**	85.71	91.32	**79.12**	**71.25**	**93.18**	**83.10**	**88.30**	**87.31**

**Table 7:** Comparison with other VPR methods at different environments in terms of R@1%.

Method (R@1%)	Type	Freiburg-A			Freiburg-B		Saarbrücken-A		Saarbrücken-B			
		Cloudy	Night	Sunny	Cloudy	Sunny	Cloudy	Night	Cloudy	Night	Sunny	Global
Siamese VGG16^55^	2D	90.17	90.17	82.12	77.59	68.73	67.90	43.10	68.90	59.43	66.97	71.51
Triplet VGG16^24^	2D	96.18	98.41	81.60	83.27	73.73	66.36	42.52	55.50	55.63	73.85	72.71
AnyLoc^20^	2D	98.69	99.22	97.92	91.19	**98.05**	95.16	92.24	99.52	91.95	98.28	96.22
SALAD^22^	2D	98.69	99.45	97.87	90.69	96.49	95.16	92.46	98.44	93.91	97.59	96.08
CosPlace^18^	2D	98.77	99.37	96.40	91.48	96.38	95.29	85.84	98.92	90.69	98.85	95.19
EigenPlaces^19^	2D	98.15	99.45	95.79	91.48	95.88	95.34	88.18	99.28	90.00	98.85	95.24
CricaVPR^56^	2D	96.15	99.00	97.92	89.59	97.22	94.24	91.84	99.88	92.87	99.20	95.79
MixVPR^21^	2D	97.53	99.48	**98.01**	89.79	96.38	94.33	90.43	99.40	90.57	**99.31**	95.52
MinkLoc3Dv2^27^	3D	96.74	99.85	96.17	91.04	96.61	97.51	94.09	98.56	90.80	97.02	95.84
CASSPR^54^	3D	97.44	99.55	97.30	91.83	94.55	98.39	92.50	98.33	91.03	95.76	95.67
**pL-MinkUNeXt (ours)**	3D	**99.11**	**99.67**	97.78	**92.48**	97.89	**98.94**	**95.31**	**100.0**	**94.71**	**99.31**	**97.52**

**Fig. 7:**
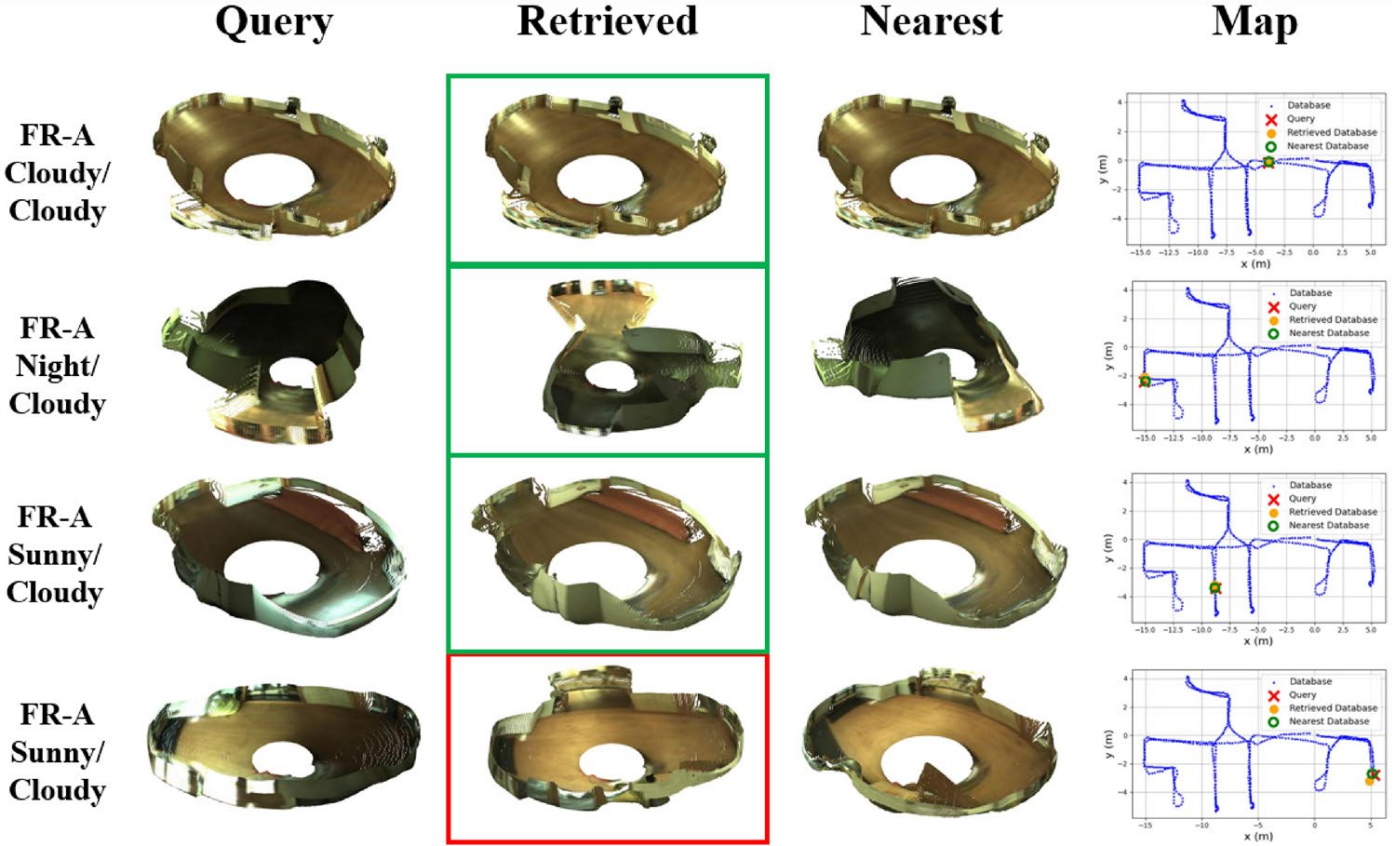
Freiburg-A. Four examples of queries and the corresponding retrieved point clouds and nearest point clouds in the database, considering different lighting conditions.

**Fig. 8:**
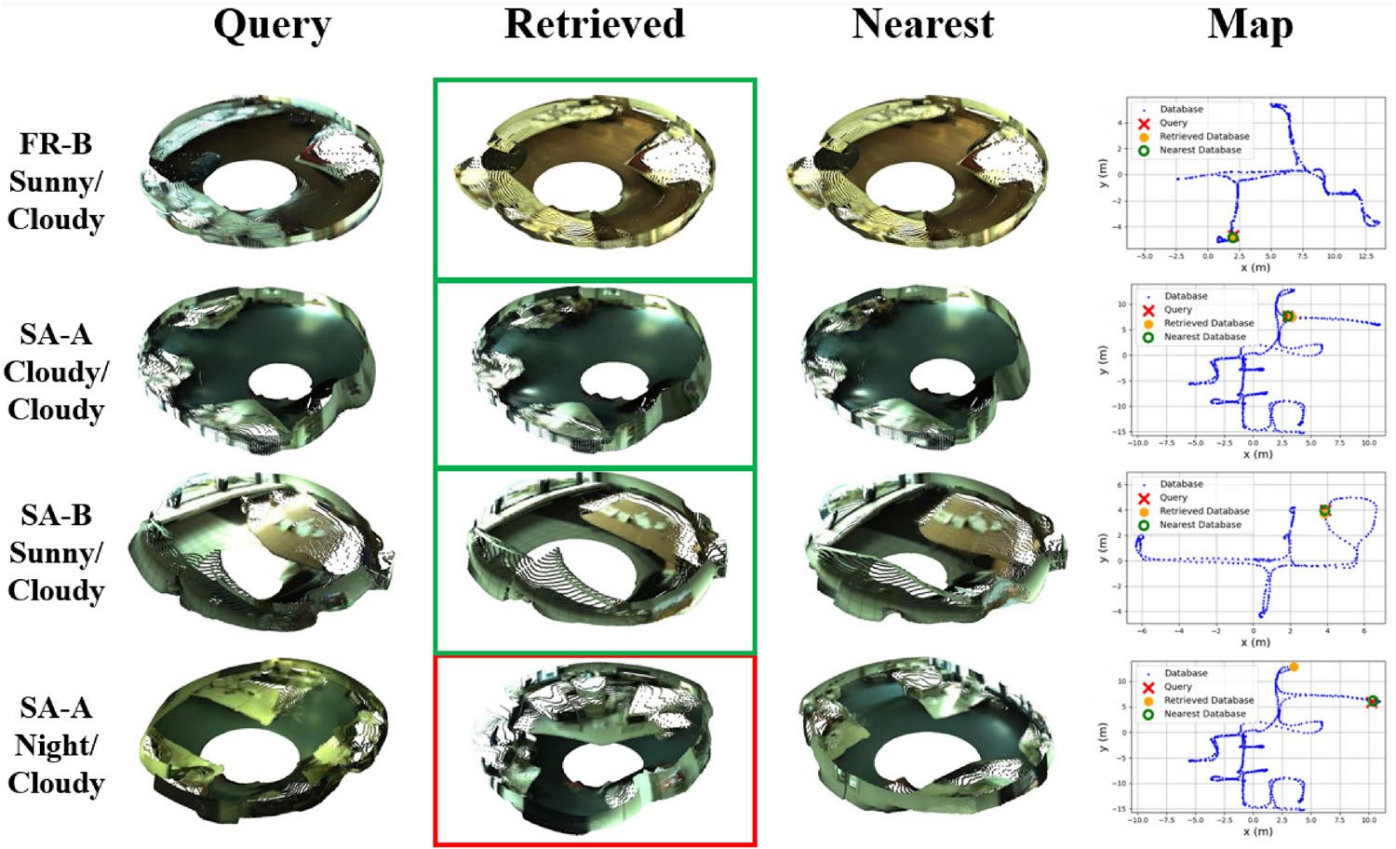
Rest of environments. Four examples of queries and the corresponding retrieved point clouds and nearest point clouds in the database, considering different lighting conditions.

In order to visually assess the performance of our method in challenging situations, Figs. 7 and 8 display several examples of queries and their corresponding nearest and retrieved point clouds, as well as a map with the positions of each of them, in different scenarios and lighting conditions.

These examples reveal that, broadly, the robot’s position was correctly retrieved in any situation, even with significant changes in illumination (FR-B sunny) and orientation (FR-A night). There were some punctual errors caused by the presence of large windows that cause reflections in the scene (FR-A sunny) or due to visual aliasing (SA-A night), which occurs when different positions of the map have a similar appearance. Nevertheless, this approach showed great robustness to changes in the appearance of the scene. To see more examples, please visit the project page (https://juanjo-cabrera.github.io/projects-pL-MinkUNeXt/).

### Generalization to other datasets

**Fig. 9:**
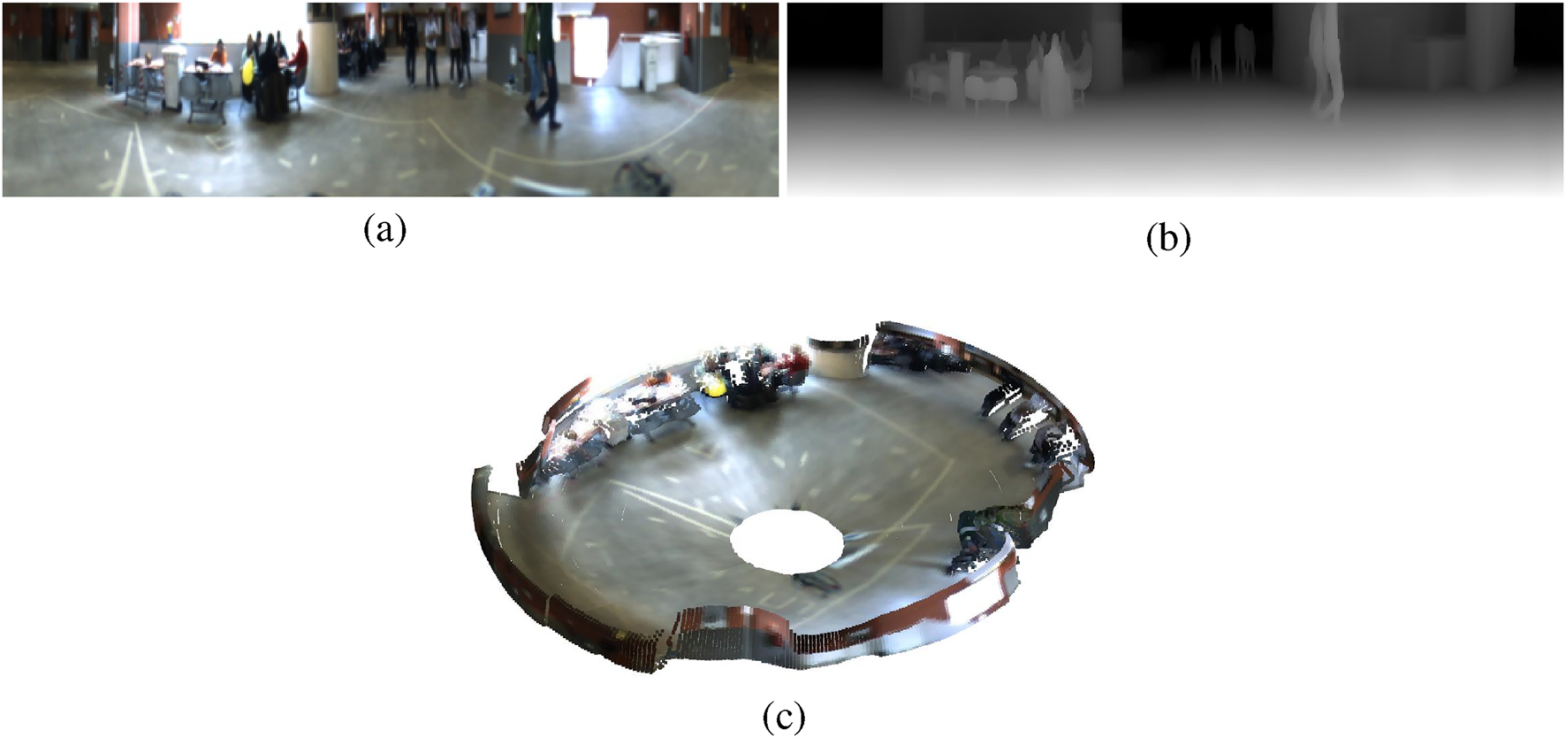
Examples of (**a**) a panoramic image from the Rawseeds dataset, (**b**) a depth map obtained with Distill Any Depth from the panoramic image and (**c**) point cloud obtained with the inverse cylindrical projection.

Finally, pL-MinkUNeXt is compared against previous state-of-the-art methods using the Rawseeds dataset^57^. This dataset contains diverse sequences captured with a catadioptric camera under day and night conditions. Specifically, the sequence Bicocca 27a (day) is employed as the reference map, whereas the sequences Bicocca 25a (day), 26a (day), 25b (night), and 26b (night) are used as queries. Figure 9 shows an example of a panoramic image from the Bicocca 25a sequence and its corresponding depth map and point cloud.

**Fig. 10:**
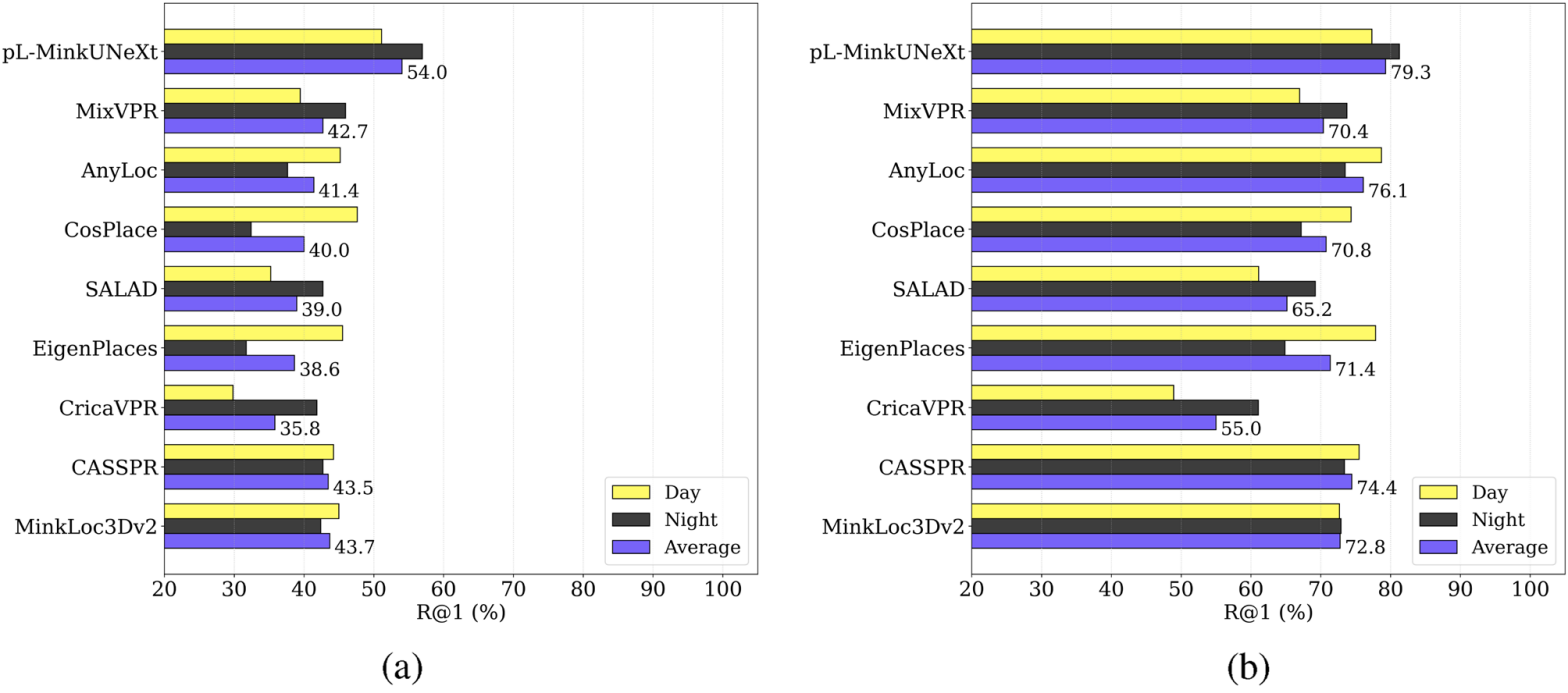
Comparison of pL-MinkUNeXt with the state-of-the-art approaches in the Rawseeds dataset, in terms of (**a**) R@1 (**b**) R@1%.

To assess the generalization capability, the model trained exclusively on the COLD dataset is evaluated directly on Rawseeds without any further fine-tuning. Figure 10 presents the R@1 and R@1% results for pL-MinkUNeXt alongside other state-of-the-art approaches.

The results demonstrate the superior generalization capability of the proposed method. In terms of Recall@1 (Fig. 10 (a)), pL-MinkUNeXt achieves the highest overall accuracy with a global average of 54.0%, significantly outperforming the second-best method, MinkLoc3Dv2^27^ (43.7%). Standard 2D methods such as CricaVPR^56^ and EigenPlaces^19^ struggle to generalize to this unseen environment, reaching only $\approx$35-39% accuracy. The most significant advantage of our pseudo-LiDAR approach is observed in the day-to-night domain shift. As shown in the detailed results, purely visual methods suffer a drastic performance drop when moving from day to night queries. In contrast, pL-MinkUNeXt exhibits remarkable robustness, even improving its performance in night scenarios compared to day scenarios. This confirms that the structural depth features distilled by our pipeline are more invariant to illumination changes than RGB descriptors, allowing the model to perform reliable place recognition in completely unseen environments.

Regarding the R@1% metric (Fig. 10 (b)), which indicates the ability to retrieve at least one correct match within the top 1% of candidates, pL-MinkUNeXt maintains its leadership with a global average of 79.3%. While some methods like AnyLoc^20^ compete closely in day scenarios, our method dominates in night conditions, proving its suitability for long-term autonomy where lighting conditions are unpredictable.

### Computational cost analysis

A critical evaluation of the method must consider not only its place recognition capabilities but also its computational efficiency, particularly for mobile robots operating in real-world applications. Table 8 presents a comparative analysis of the inference time and GPU memory usage for each module of the proposed pipeline. Regarding individual components, the pL-MinkUNeXt architecture incurs the highest computational cost, yet it remains efficient enough for high-frequency loops. Conversely, the Distill Any Depth model is the most memory-intensive component (1.3 GB), although it is temporally efficient (10.2 ms). Meanwhile, the gradient estimation and cylindrical projection entail negligible computational overhead. Finally, the total inference time of 30.2 ms and the VRAM requirement of 2.0 GB make the system suitable for embedded platforms (e.g., NVIDIA Jetson series), facilitating its deployment on resource-constrained mobile robots. Consequently, the proposed method is fully capable of real-time operation, leaving sufficient temporal margin for simultaneous tasks such as path planning or obstacle avoidance.

**Table 8:** Inference time and GPU memory consumption of each module in our proposed pipeline.

Module	Gradient Estimation	Distill Any Depth (Large)	Cylindrical Projection	pL-MinkUNeXt	Total
Time [ms]	0.1	10.2	4.0	15.9	30.2
GPU Mem. [GB]	-	1.3	-	0.7	2.0

## Conclusion

In this manuscript, a method to tackle place recognition using pseudo-LiDAR point clouds derived from panoramic images is presented. To create these point clouds, a depth map is generated from every panoramic image using Distill Any Depth, a state-of-the-art depth estimator that exploits the benefits of distillation. The resulting pseudo-LiDAR information is then fed to a MinkUNeXt model to compute global-appearance descriptors. Despite the fact that vision sensors are greatly affected by natural changes in illumination, our approach proved a great ability to increase the invariance of descriptors across dissimilar environments and illumination conditions, presenting itself as an effective solution for place recognition.

Besides, a novel data augmentation technique is proposed, which consists in training the network with different depth estimators, i.e. Distill Any Depth and DAv2, along with their distilled versions (Small, Base, Large). Additionally, point clouds are enriched with visual features based on the intensity gradient. In summary, the proposed approach demonstrates excellent performance in place recognition due to the combination of point clouds and efficient data augmentation strategies, based on the use of distilled depth estimators.

This research work demonstrated the versatility of the proposed approach, validating its effectiveness in new environments and on both omnidirectional and standard pinhole cameras. Having established this sensor flexibility, future research will aim to incorporate semantic visual information alongside the depth estimation techniques. We plan to explore early and late fusion strategies to combine these modalities, thereby improving the descriptive power of the descriptors for diverse robotic navigation scenarios.

## Data Availability

Data is publicly available on the project website: https://juanjo-cabrera.github.io/projects-pL-MinkUNeXt/.
